# Esophageal Endoscopic Submucosal Dissection in Older Patients Is Safe and Feasible: A Retrospective Single-Center Cohort Study in the United States

**DOI:** 10.3390/jcm13010228

**Published:** 2023-12-30

**Authors:** Mako Koseki, Makoto Nishimura, Jacques C. Beauvais, Tarek Nammour, Sayaka Nagao, Mark A. Schattner

**Affiliations:** 1 Gastroenterology, Hepatology, and Nutrition Service, Memorial Slone Kettering Cancer Center, New York, NY 10065, USA; 2 Internal Medicine, Mount Sinai Beth Israel, New York, NY 10003, USA

**Keywords:** aging, United States, endoscopic submucosal dissection, esophageal neoplasms

## Abstract

Background: Endoscopic submucosal dissection (ESD) is a well–established method for treating early esophageal carcinomas. However, data on the safety and efficacy of esophageal ESD in older patients in the United States are limited. Methods: This retrospective study investigated the outcomes of esophageal ESD in patients aged ≥80 years and included those who underwent esophageal ESD between June 2018 and April 2023 at a single center in the United States. Patients were divided into two age groups for comparison: ≥80 and <80 years. Treatment outcomes and complications were evaluated and compared between these groups. Results: A total of 53 cases of esophageal ESD for malignant neoplasms were included, with 12 patients in the ≥80 years age group. No significant differences were observed in the patients’ background and characteristics, except for a prior history of interventions (*p* = 0.04). The en bloc resection rate was 100% in both groups. The R0 resection rate was lower in the ≥80 years age group (75% vs. 88%). There were no complications requiring additional intervention in the ≥80 years age group, such as post–ESD bleeding, perforation, mediastinal emphysema, or pneumonia. Conclusions: Esophageal ESD may be a safe and feasible procedure for treating esophageal carcinomas in older patients.

## 1. Introduction

In the United States, esophageal cancer accounts for 1% of all diagnosed cancers and is the seventh most common cause of cancer–related death in men [1,2]. Approximately 16,000 esophageal cancer–related deaths occur annually in the U.S. The 5-year relative survival rate of esophageal cancer in the United States is 21%, which has improved from 5% 50 years ago due to improved treatment options [2]. However, it remains much lower than that of other gastrointestinal cancers. One reason for this is that surgical esophageal resection is associated with significant morbidity and mortality and is one of the highest-acuity procedures performed routinely in North America [1]. Therefore, esophageal cancer should be diagnosed at an early stage without metastasis and should be treated using minimally invasive approaches, including endoscopic procedures.

Endoscopic submucosal dissection (ESD) is an approach that is used for minimally invasive cancer excision. Its role has been supported by recent professional society guidelines from Asia and Europe [3,4]. In the United States, according to the recent American Gastroenterological Association (AGA) Clinical Practice Update, ESD is recognized as a mature endoscopic technique that enables en bloc resection of lesions that are too large for endoscopic mucosal resection (EMR) or that are at an increased risk of cancer [5]. Furthermore, a guideline on ESD for the management of early esophageal and gastric cancers was recently published for the first time in the United States by the American Society for Gastrointestinal Endoscopy (ASGE) [6]. Therefore, ESD is a safe and effective treatment for esophageal carcinoma. However, reports on ESD for esophageal cancers in the United States are limited, as it is mainly performed at high–volume centers, even after its feasibility and safety have been proven [7]. ESD has become an important tool for the management of early lesions in the United States.

With the aging society, endoscopic procedures for cancer resection have rapidly gained popularity. Esophageal ESD is increasingly performed worldwide, even in older adults, because it is less invasive and has fewer procedural and postoperative risks than open surgery. It also enhances the postprocedural quality of life. However, no guidelines state its age limitations, and limited data exist on the safety and efficacy of esophageal ESD in older patients in this country.

Therefore, this study aimed to investigate the outcomes of esophageal ESD in patients aged ≥80 years, which is considered an extreme age, to provide supportive evidence on this procedure as an option for the treatment of older adults in the United States.

## 2. Materials and Methods

### 2.1. Study Population

This single-center retrospective study conducted in the United States was approved by the Institutional Review Board (Memorial Sloan Kettering Cancer Center 20-495 A [2]), and informed consent was obtained from all patients for ESD procedures. We retrospectively reviewed the data of all patients who underwent ESD for esophageal cancer between June 2018 and April 2023 at our facility. This study defined patients aged ≥80 years as older adults. A total of 53 esophageal ESDs were performed for adenocarcinoma and squamous cell carcinoma during the study period, with 12 cases involving patients aged ≥80 years. The indications for ESD were as follows: (i) known malignancy with no lymph node or distant metastasis on imaging studies and (ii) tumor limited to the mucosa or submucosa on observation.

### 2.2. Evaluation of Baseline Patient Characteristics

We assessed the patients’ baseline demographic and clinical characteristics, including age, sex, use of anticoagulation and antiplatelet medications, and history of esophageal intervention. Prior esophageal interventions included radiofrequency ablation, radiation, EMR, and ESD. Additionally, we assessed the type of anesthesia used during the procedure using the American Society of Anesthesiologists (ASA) score.

### 2.3. Perioperative Details

The patients were instructed to refrain from oral food intake at midnight on the day of the procedure. Anticoagulation medications were stopped according to the ASGE guideline [8]. The patients presented to our facility on the day of the procedure and were hospitalized for one or two nights following the procedure. If no signs of complications were observed, patients were allowed to consume a clear liquid diet on the same day. The following day, after confirmation of stable blood test results, a full liquid diet was allowed, and patients were discharged if they could tolerate food intake well. If signs of bleeding, including a drastic drop in hemoglobin levels in blood tests or any other irregular data, were observed, patients were recommended to stay in the hospital until their condition stabilized.

### 2.4. Endoscopic Submucosal Dissection Procedure

Endoscopic resection was performed under general anesthesia administered via endotracheal intubation in the endoscopy unit. All ESD procedures were performed by an experienced endoscopist (M.N.) using a standard upper endoscope (GIF-H190, Olympus America, Center Valley, PA, USA). At this time, the endoscopist (M.N.) performed approximately 1,700 ESD procedures and had 21 years of advanced endoscopy experience, including 20 peroral endoscopic myotomy procedures. At our facility, approximately 150–200 ESD procedures are annually performed in the endoscopic unit by the endoscopist.

Submucosal injections were performed using ORISE gel (Boston Scientific, Marlborough, MA, USA; no longer available because of recall), Eleview (Medtronic, Louisville, CO, USA), Blue Eye (Omnimed, Winchester, UK), or EndoClot SIS (Olympus America). For cutting, a needle–type knife, either a 1.5-mm or 2.0-mm ORISE ProKnife (Boston Scientific) or DualKnife J (Olympus America), was used. The ESD method was consistent in all the cases. First, mucosal markings were made at 2 mm intervals around the lesion using a needle–type knife. Submucosal injection of a lifting agent was then performed to elevate the lesion. Mucosal incisions were made using a needle–type knife, and the submucosal layers were dissected to remove the lesion. If necessary, submucosal injection using an injection needle or through the knife was repeated during the procedure. Endoscopic hemostasis was achieved using a Coagrapser (Olympus, America). Carbon dioxide insufflation was performed during the procedure. Specimens were extracted orally and placed in formalin.

### 2.5. Histological Assessment

For pathological evaluation, all resected specimens were fixed in formalin and sectioned to assess tumor involvement. All the resected specimens were evaluated by an experienced pathologist specializing in gastroenterology. Unlike some Eastern Asian countries where specific guidelines are available for histologic classification, there are no guidelines in the U.S. for histologic classification of neoplasms removed specifically by endoscopic procedures. Therefore, pathologists used the WHO classification of tumors for the pathological evaluation. We evaluated the en bloc resection rate, R0 resection rate, pathological diagnosis, tumor size, tumor depth, lymphovascular involvement, and lateral and deep margins.

En bloc resection was defined as a one–piece resection. R0 resection was defined as an en bloc resection with histologically confirmed tumor–free margins. Basement membrane tumors (pTis), intramucosal tumors (pT1a), and submucosal tumors (pT1b) were identified.

### 2.6. Adverse Events

The number of hospitalization days and complications were compared between the groups. Hospitalization days were defined as the number of nights the patient stayed at the hospital. All procedural complications were extracted from the medical charts. Post-ESD bleeding was defined as bleeding that occurred after the ESD procedure at any time and necessitated another endoscopy or a hemoglobin drop of at least 1.0 (g/dL) within 24 h. A perforation was defined as a deep defect in the muscularis propria with or without direct contact with the connective tissue outside the esophagus that developed during or after the ESD procedure. Strictures were defined as those requiring further intervention.

### 2.7. Follow-Up

Post-treatment surveillance for recurrence was performed in all the patients. Endoscopic examination and evaluation were performed 3–6 months after ESD, depending on the lesion size, pathological results, and patient preference. Patients with adenocarcinoma were mainly followed up every 3 months until 1 year after ESD, whereas patients with squamous cell carcinoma were followed–up every 6 months until 1 year after ESD. After 1 year, patients with both types of cancer were followed–up annually unless they had high–risk factors. Cases involving positive margins in ESD were discussed by the institutional tumor board and referred to a surgeon or oncologist as needed to consider surgical or oncological approaches. Cross–sectional imaging, such as computed tomography and positron emission tomography, was considered and performed in most cases with risk factors for lymph node metastasis, such as positive lymphovascular invasion. When positive margins were observed but surgery or oncological treatment was not feasible, alternative interventions such as brachytherapy or cryotherapy were discussed, and the patients were closely monitored for any signs of recurrence.

### 2.8. Statistical Analysis

Categorical variables were compared between the two groups using Pearson’s chi-square or Fisher’s exact test. Continuous variables were compared using interquartile ranges. The hazard ratios (HRs) and 95% confidence intervals were calculated. Descriptive statistics were calculated using the median and interquartile ranges for continuous variables.

## 3. Results

### 3.1. Patient Characteristics

A total of 53 cases involving esophageal ESD for malignant neoplasms were included in this study for statistical analysis, with 12 cases in the ≥80 years age group (23% of the total number). The baseline characteristics of the study population are presented in Table 1. There were no significant differences in the patient background characteristics between the two groups. The oldest patient who underwent ESD was a 96-year-old male. Patients in the ≥80 years age group had a significantly higher history of interventions before ESD than those in the younger age group (*p* = 0.04). Six patients in the ≥80 years age group had a history of EMR, and among those, 4 had a prior history of radiofrequency ablation (RFA). Furthermore, 1 patient had a history of chemoradiotherapy. In the <80 years age group, 2 patients had a history of EMR, 3 had a history of RFA, and 5 had a history of chemoradiation therapy.

### 3.2. Endoscopic Procedure

The median procedure time (interquartile range (IQR)) was 100 (63–150) minutes in the ≥80 years age group and 90 (65–108) minutes in the <80 years age group (*p* = 0.93). The specimen size was also similar, with a median of 35 mm in both the groups. The locations of the lesion were dispersed. The characteristics of the lesions are shown in Table 2.

### 3.3. Histology

A total of 53 lesions were evaluated by the pathologists (Table 3). More than 75% of the cases were adenocarcinomas, and the rest were squamous cell carcinomas. The en bloc resection rate was 100% in both groups. The R0 resection rate was 75% in the ≥80 years age group compared to 88% in the <80 years age group (*p* = 0.36). There were no significant differences in the lateral margin, deep margin, or lymphovascular invasion.

### 3.4. Complications

Complications are shown in Table 4. The median hospitalization period (IQR, days) was 1 (1-1) vs. 1 (1-2) (*p* = 0.10). No complications were observed in patients aged ≥80 years, whereas two patients had complications in the <80 years age group. One patient was treated on the day of the procedure, a repeat endoscopy was performed, and clipping was performed to control bleeding from the oozing artery. Another patient required rehospitalization because bleeding occurred 6 days after ESD. Repeat endoscopy was performed, but no active bleeding was observed. One patient in the <80 years age group experienced perforation during the procedure, where the clip was placed with conservative management and required 3 days of hospitalization. Esophageal strictures were seen as a delayed complication in 8.3% (n = 1/12) and 10% (n = 4/41) (*p* = 1.00) of patients in the ≥80 and <80 years age groups, with median lesion sizes of 90 mm and 55 mm, respectively. All patients had circumferential lesions larger than one–third of the esophageal circumference and were endoscopically treated with multiple balloon dilatations and steroid injections. One patient required a stent placement. There were no deaths related to ESD.

### 3.5. Follow-Up

Seven out of 12 patients in the ≥80 years age group and 36 out of 41 patients in the <80 years age group underwent repeat endoscopy surveillance at our center, either 3 or 6 months after ESD. Two patients experienced a recurrence of malignancy in both groups, although all of these lesions were at a different location from that of lesions treated by ESD. No recurrence occurred at the ESD location. Moreover, 7 patients in the <80 years age group and none in the ≥80 years age group underwent esophagectomy for non-R0 resections after ESD.

## 4. Discussion

To our knowledge, this is the first study to demonstrate the feasibility and safety of esophageal ESD in older adults in North America. Our results indicate that ESD can be considered as an option for early esophageal cancer in older adults, as there were no significant differences in the histological results and complication rates between patients aged ≥80 years and those aged <80 years. Moreover, recurrence was not observed at the same location in any of the cases.

Esophageal cancer is characterized by a poor prognosis, necessitating early treatment. With the increasing prevalence of ESD in the United States, the demand for this procedure is growing. Many older adults maintain good health despite age, and although they may have comorbidities, they have longer–than–average life expectancy. ESD is a viable option for these patients despite having more comorbidities, poorer physical status, and shorter life expectancies than younger patients.

Esophagectomy provides the best chance of cure for all stages, including the early stages. However, it is a technically demanding and invasive procedure with the potential for high rates of short–term mortality and morbidity ranging from 7% to 13% [9]. Older patients are at even greater risk. One report demonstrated that short–term risks outweighed the benefits of the long–term cure offered by surgical resection. It was concluded that endoscopic treatment is a reasonable approach for early esophageal cancers in older adults [10]. Thus, ESD should be considered the first-line treatment for early esophageal malignancy in older patients.

The ASGE guidelines on ESD for early esophageal cancers were published in the United States in 2023 [6]. As already suggested in the AGA Clinical Practice Update, which was published before the ASGE guidelines, the indications for ESD for esophageal cancer differ between squamous cell carcinoma (SCC) and adenocarcinoma [5].

For SCC, a lesion limited to m1/m2 in the mucosal layer (m1, intraepithelial non–invasive carcinoma: m2, carcinoma invading the lamina propria) with involvement of 2/3 or less of the esophageal circumference is an indication for ESD, whereas a lesion progressing to m3 (defined as carcinoma extending to or invading the muscularis mucosa with <200 microns of invasion into the submucosa) with a clinical N0 status (no cancer detected in regional lymph nodes) represents an expanded indication. With regard to size, the ASGE recommends ESD over EMR for well–differentiated nonulcerated cancers measuring >15 mm and either ESD or EMR for lesions measuring ≤15 mm. Our study included an 87-year-old patient with a history of multiple heart surgeries and a clinical T1b SCC (submucosal invasion). The patient declined surgery and successfully underwent ESD without complications. Although the lateral margin was positive, the deep margin and lymphovascular invasion were negative in pathological analyses. Follow–up data were not available because the patient returned to her home country after ESD.

For adenocarcinoma, a cT1a (clinically only in the mucosal layer), a well–differentiated, nonulcerated lesion, is a suggested indication for ESD. With regard to size, the ASGE recommends ESD over EMR for lesions measuring >20 mm and either ESD or EMR for lesions measuring ≤20 mm. Most patients in the ≥80 years age group who underwent ESD for adenocarcinoma in our study had previously undergone EMR with positive margins. These patients underwent multiple EMRs and RFAs for Barrett’s esophagus. Despite these interventions, malignancy ultimately develops, necessitating ESD. Notably, in our study, ESD was performed in a few cases that did not strictly meet the suggested indications for patients aged ≥80 years. Many of these patients underwent ESD because they were not suitable candidates for surgery, even if the likelihood of curative resection was low based on the preoperative criteria. However, it is crucial to emphasize that all ESD procedures were intended for R0 resection, and none were performed as palliative measures.

Feasibility and effectiveness are fundamental considerations when evaluating ESD. Our study achieved a 100% en bloc resection rate in both age groups. The R0 resection rate was similar to that in previous studies from Asia and Europe, where the R0 rate typically ranged from 70% to 90% [11,12,13].

The median age to perform esophageal ESD for esophageal cancer is approximately 65 years in East Asian countries, per previous reports [14,15]. The median age in the present study was 71 years, which is slightly higher than that reported in previous studies. However, the median age at diagnosis of esophageal cancer in our country is 68 years, with most patients diagnosed between 65 and 74 years of age; therefore, the age of our study population was considered appropriate [16]. Furthermore, the above studies have compared the outcomes of younger and older age groups in other countries, and the age of their study patients ranged from the 40s to the 90s. A similar age distribution of 47 to 94 years was observed in the present study.

ESD in the esophagus presents challenges due to the narrow lumen and thin wall of the esophagus, which continuously moves with respiration and cardiac pulsations, making endoscopic procedures more difficult [17]. Therefore, patients occasionally develop complications such as perforation and bleeding [18]. In our study, there were no significant differences in the incidence of adverse events between the older and younger groups. All procedural complications in our study occurred in younger age groups. Strictures occurred in 4 cases in the younger group and 1 case in the older group, all of which required further endoscopic interventions. Three lesions with strictures in the middle third of the esophagus had resection sizes of 90, 60, and 60 cm, one lesion in the lower third of 50 cm, and one in the gastroesophageal junction with a 25-cm lesion. All lesions were circumferential in shape. We believe stricture development is occasionally inevitable in patients with large lesions.

Prior endoscopic therapies such as EMR, RFA, and chemoradiation therapy often result in submucosal fibrosis, which can complicate subsequent endoscopic therapies [19]. In particular, many patients in the United States have a history of multiple previous interventions, given the higher rate of adenocarcinoma compared with that in East Asian countries. Generally, older patients undergo more procedures for Barrett’s lesions because they have been receiving treatment for a longer period of time. Our study identified a significant difference in the history of prior interventions between the two age groups. Such interventions make the subsequent procedures more difficult. Furthermore, depending on their age and comorbidities, ESD is strongly recommended by surgeons for older patients at our institution. Multiple patients with cT1b lesions underwent ESD. Even with positive margins, these lesions could be treated with additional local control therapies such as cryotherapy or brachytherapy in the U.S. Despite these differences, the R0 resection rates for our patients were similar to those in previous reports. Moreover, ESD was successfully performed without complications in all older patients, underscoring its safety.

The length of hospital stay did not differ significantly between the two age groups, a finding consistent with those of studies conducted in Japan [15,20]. These results suggest that ESD can be safely performed in older patients without exacerbating chronic conditions [21]. Two previous reports from China have shown that the natural history of early esophageal SCC takes a long time to progress from early to advanced stages, with >50% of untreated patients surviving for 5 years after diagnosis and the remaining progressing to death within the same time period [22,23]. However, the prognosis of early esophageal adenocarcinoma originating from Barrett’s esophagus remains unclear because most patients are treated for dysplasia before progressing to cancer, and there are no published reports on untreated adenocarcinoma related to Barrett’s esophagus. However, the progression of Barrett’s dysplasia to cancer occasionally takes a long time [24]. Taken together, patients have the choice to leave early esophageal cancer untreated and take the risk of progression. Thus, even though endoscopic procedures are less invasive than surgery, older patients with a high risk (especially ASA > 4) for ESD should be educated about the prognosis of their disease and the possibility that they may or may not live for a few years with or without treatment.

Although our study provides valuable insights into the feasibility and safety of ESD for early esophageal cancer in older patients, it is not without its limitations.

One limitation is that we have not been able to acquire long–term outcomes, as our center started performing ESD 5 years ago and has more recently been performing ESDs in older patients. Additionally, the follow–up rate with endoscopy in the ≥80 age group was not sufficiently high for robust intergroup comparisons. Continuous monitoring with an annual focus on recurrence rates is necessary to address this limitation.

Another limitation was that our study had a single–center design in which a single endoscopist performed all ESD procedures. Furthermore, the number of included cases was relatively too small to achieve statistical power and conclusions; this was a major limitation. Although ESD has gained traction in the United States, the number of procedures performed remains limited nationwide. As a renowned cancer center, our institution may attract patients with better access to resources and higher health literacy, potentially resulting in fewer comorbidities. However, these findings mirror the reality of clinical practice in the United States.

## 5. Conclusions

In conclusion, our study demonstrated no significant differences in the short–term outcomes of esophageal ESD for early esophageal cancer between older and younger age groups. Therefore, esophageal ESD is a feasible and safe treatment for esophageal carcinomas in elderly patients. However, further research, including long–term follow–up, is warranted to validate the effectiveness of this approach in the United States.

## Figures and Tables

**Table 1:** Patient Characteristics.

	Age ≥ 80 Years	Age < 80 Years	*p* Value
Number of patients	12	41	
Median age, years (range)	83 (80–96)	69 (47–78)	<0.001
Sex			0.15
Male	6 (50%)	31 (76%)	
Female	6 (50%)	10 (24%)	
History of prior interventions	7 (58%)	10 (24%)	0.04
Anticoagulation	3 (25%)	4 (10%)	0.18
American Society of Anesthesiologists score			0.10
2	0	9 (22%)	
3	10 (83%)	30 (73%)	
4	2 (17%)	2 (5%)	

**Table 2:** Lesion Characteristics.

	Age ≥ 80 Years	Age < 80 Years	*p* Value
Location			0.54
Upper	2 (17%)	2 (5%)	
Mid	4 (33%)	19 (48%)	
Lower	5 (42%)	10 (25%)	
GEJ	1 (8%)	9 (23%)	
ESD time (minutes)	100 (63–150)	90 (65–108)	0.93
Specimen size (mm)	35 (34–43]	35 (30–40)	0.55

GEJ: gastroesophageal junction, ESD: endoscopic submucosal dissection.

**Table 3:** Histological Results.

	Age ≥80 Years	Age <80 Years	*p* Value
En bloc resection	12 (100%)	41 (100%)	
R0 resection	9 (75%)	36 (88%)	0.36
Diagnosis			1.00
AdenoCa	9 (75%)	31 (76%)	
SCC	3 (25%)	10 (24%)	
Cancer-Depth			0.94
pTis	1 (8%)	4 (10%)	
pT1a	6(54%)	19 (46%)	
pT1b	5 (39%)	18 (44%)	
Cancer-LM			0.22
+	2 (17%)	2 (5%)	
−	10 (83%)	39 (95%)	
Cancer-VM			1.00
+	1 (8%)	3 (7%)	
−	11 (92%)	38 (93%)	
Cancer-LVI			1.00
+	2 (17%)	8 (20%)	
−	10 (83%)	33 (80%)	

AdenoCa, adenocarcinoma; SCC, squamous cell carcinoma; LM, lateral margin; VM, deep margin (vertical margin); LVI, lymphovascular invasion.

**Table 4:** Complications.

	Age ≥80 Years	Age <80 Years	*p* Value
Hospitalized days	1 (1-1)	1 (1-2)	0.13
Post-ESD bleeding	0	2 (5%)	1.00
Esophageal perforation	0	1 (2%)	1.00
Stricture	1 (8%)	4 (10%)	1.00

## Data Availability

Data are contained within the article.
